# Serglycin modulates inflammation and metabolism in macrophages

**DOI:** 10.1016/j.isci.2026.115235

**Published:** 2026-03-10

**Authors:** Shirin Porteymour, Saikat Das Sajib, Susannah von Hofsten, Atanaska I. Doncheva, Christina D. Bjørnvall, Beate Hegge, Mélina Gautier, Mirjana Grujic, Pratibha Kolan, Karthickeyan Chella Krishnan, Jason Matthews, Knut T. Dalen, Gunnar Pejler, Svein O. Kolset, Erik Knutsen, Frode A. Norheim

**Affiliations:** 1 Department of Nutrition, Institute of Basic Medical Sciences, University of Oslo, Oslo, Norway; 2 Department of Medical Biology, Faculty of Health Sciences, UiT The Arctic University of Norway, Tromsø, Norway; 3 Department of Medical Biochemistry and Microbiology, Uppsala University, Uppsala, Sweden; 4 Department of Pharmacology, Physiology, and Neurobiology, University of Cincinnati, College of Medicine, Cincinnati, OH, USA; 5 Department of Pharmacology and Toxicology, Temerty Faculty of Medicine, University of Toronto, Toronto, ON, Canada

**Keywords:** immunology, cell biology, molecular biology

## Abstract

Serglycin is a key hematopoietic proteoglycan that regulates the storage and retention of proteases, cytokines, and chemokines within immune-cell secretory granules. We previously identified serglycin as the predominant proteoglycan secreted by macrophages and a regulator of tumor necrosis factor-α (TNF-α) release following lipopolysaccharide (LPS) stimulation. Here, we analyzed serglycin expression in peritoneal macrophages from 92 mouse strains in the hybrid mouse diversity panel (HMDP) after LPS exposure and investigated its role in M1 polarization using serglycin-deficient murine bone marrow-derived macrophages and human THP-1 macrophages stimulated with LPS and interferon-γ (IFN-γ). Serglycin upregulation is strongly associated with M1 polarization and correlated with TNF-α and IFN-γ transcriptional signatures across models. Integrated transcriptomic and secretome analyses revealed a marked discordance between mRNA and protein output in serglycin-deficient macrophages, with impaired cytokine secretion despite elevated transcript levels, validated by RT-qPCR and ELISA. Loss of serglycin additionally affected macrophage metabolism, vesicle biogenesis, phagocytosis, and extracellular reactive oxygen species generation, identifying serglycin as a key regulator of macrophage polarization and functional programming.

## Highlights

• Serglycin induction is associated with M1 macrophage polarization in mouse and human models
• Serglycin deficiency uncouples cytokine transcription from secretion in activated macrophages
• Serglycin regulates macrophage metabolism, vesicle biogenesis, and effector functions

## Graphical abstract

**Figure:**
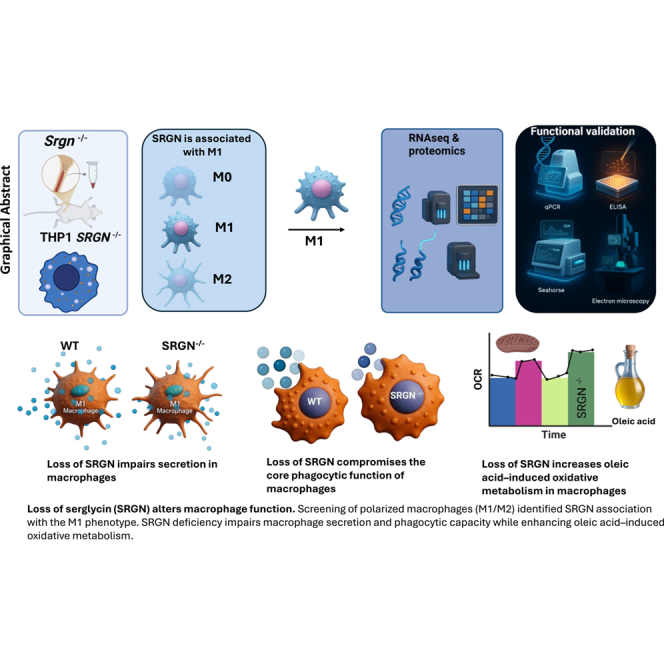

## Introduction

Proteoglycans consist of diverse core proteins covalently attached to sulphated glycosaminoglycans (GAGs) of different compositions. Among proteoglycan species, serglycin is highly expressed in hematopoietic cells and is also found in several non-hematopoietic cell types, including cancer cells.^1^^,^^2^ The importance of serglycin for the proper functions of many immune cells, including mast cells, macrophages, and neutrophils, is well established. Serglycin is stored in secretory granules and released in response to cell-specific stimuli.^3^ In mast cells, serglycin is essential for storing granule constituents, such as histamine and proteases, and is released upon the crosslinking of IgE receptors during allergic responses.^4^ Serglycin also exerts important functions in other immune cell types. For example, serglycin regulates tumor necrosis factor-alpha (TNF-α) secretion in lipopolysaccharide (LPS)-activated macrophages.^5^ Moreover, serglycin promotes granzyme B storage in cytotoxic T cells,^6^ and elastase storage in neutrophils, the latter with consequences for antibacterial defense.^7^

The functional role of serglycin is cell-specific and largely determined by the type of GAG chains covalently attached to its core protein. In connective tissue-type mast cells, serglycin carries heparin-type GAG chains. Heparin is highly sulphated, imparting a strong negative charge. This property contributes to its potency as a widely used clinical anticoagulant. In addition to heparin, mast cells and most other immune cells attach chondroitin 4-sulphate GAG chains to serglycin. Notably, activated monocytes and macrophages can also synthesize oversulphated chondroitin sulfate (chondroitin 4,6-disulphate) on serglycin.^3^ The type and degree of GAG sulphation on serglycin critically influence its capacity to interact with partner molecules, thereby shaping its biological functions.

In inflammatory responses, the interactions between serglycin and its partner molecules are functionally critical. Serglycin binds to chemokines and serine proteases, modulating their stability, localization, and biological activities.^8^^,^^9^^,^^10^ Beyond serving as a granule-retention scaffold, serglycin facilitates regulated secretion, extracellular transport, and the protection and presentation of molecular partners to target cells. Together, these functions position serglycin as a key regulator of immune cell effector mechanisms during inflammation.

Recent studies have expanded knowledge of the role of serglycin in health and disease. In serglycin knockout mice, aged animals displayed enlarged lymphoid organs, including spleens and Peyer’s Patches.^11^ In a 20-week high-fat, high cholesterol diet study, serglycin protected against diet-induced increases in serum low-density lipoprotein.^12^ In human monocytes, *in vitro* LPS stimulation markedly increased the secretion of inflammatory mediators and expanded the number and size of serglycin-containing vesicles.^13^ Moreover, emerging evidence implicates macrophage-derived serglycin in the pathophysiology of inflammatory diseases, such as osteoarthritis,^14^ post-stroke neuroinflammation,^15^ and intervertebral disc degeneration.^16^ Notably, in an eight-week high-fat and high-sucrose diet intervention, serglycin knock-out mice exhibited fewer M1 macrophages in adipose tissue than wild-type mice.^17^ The latter findings thus raise the possibility that serglycin can be associated with processes involving macrophage polarization. Here, we examined this hypothesis using complementary approaches, including studies in which wild-type and serglycin-deficient murine (bone marrow-derived) and human (THP-1) macrophage-like cells were subjected to phenotypic polarization and activation by bacterial LPS and IFN-γ. Additionally, we profiled serglycin expression in LPS-stimulated peritoneal macrophages derived from 92 mouse strains of the hybrid mouse diversity panel (HMDP). Our results reveal a strong association between serglycin and macrophage polarization, demonstrating its impact on the inflammatory phenotype of activated macrophages.

## Results

### Serglycin is induced by LPS stimulation and is associated with the inflammatory response and metabolism in classically activated murine macrophages

**Figure 1:**
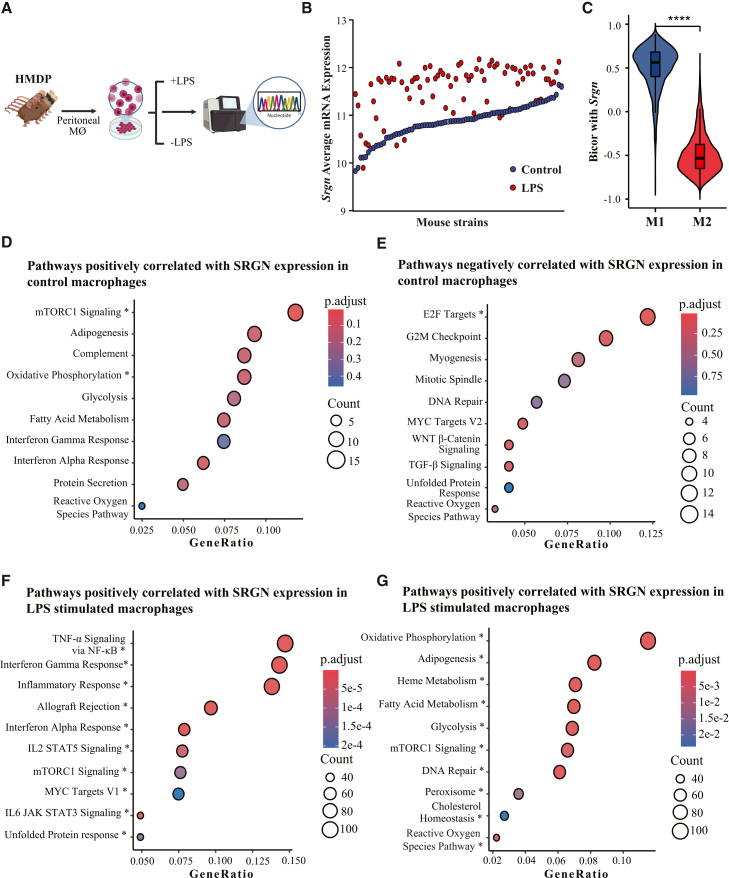
SRGN expression associates with activation of inflammatory pathways and suppression of metabolic and proliferative programs in macrophages (A) Schematic overview of experimental design. Peritoneal macrophages were isolated from 92 inbred strains of the hybrid mouse diversity panel (HMDP) and profiled by microarray analysis under basal conditions and after 24 h of LPS stimulation. (B) Basal *Srgn* expression was high across strains and was further induced following LPS stimulation, with >2-fold induction observed in ∼32% of strains. (C) Correlation of *Srgn* expression with macrophage polarization markers. *Srgn* expression correlated positively with M1 (pro-inflammatory) markers and negatively with M2 (anti-inflammatory) markers (unpaired Student’s t test; ∗∗*p* < 0.0001). (D and E) Pathway enrichment analysis of *Srgn*-correlated genes in unstimulated macrophages, with significant pathways marked by an asterisk (∗). Positively correlated genes were enriched for mTORC1 signaling, adipogenesis, oxidative phosphorylation, glycolysis, and fatty acid metabolism (D), whereas negatively correlated genes were enriched for cell-cycle-related pathways, including E2F targets, G2M checkpoint, mitotic spindle, DNA repair, and MYC targets (E). (F and G) Pathway enrichment analysis of *Srgn*-correlated genes in LPS-stimulated macrophages. *Srgn* expression correlated positively with pro-inflammatory pathways, including TNF-α signaling via NF-κB, interferon-γ/α responses, inflammatory response, allograft rejection, and IL6/JAK/STAT3 signaling (F), while negative correlations were observed with mitochondrial and lipid metabolic pathways, including oxidative phosphorylation, heme metabolism, fatty acid metabolism, peroxisome activity, and cholesterol homeostasis (G).

Macrophages are central to inflammation and are activated by the Toll-like receptor 4 (TLR4) ligand LPS, triggering pro-inflammatory cytokine release.^18^ This response enables clearance of unwanted material and recruitment of immune cells. Serglycin (SRGN) regulates the storage and secretion of inflammatory mediators in immune cells, including macrophages.^5^ To investigate further the role of serglycin in macrophage inflammation, we analyzed genome-wide transcription profiles of peritoneal macrophages from 92 mouse strains in the HMDP, under basal conditions and 24 h post-LPS stimulation. (Figure 1A). Basal *Srgn* expression was high in all strains but showed a varied expression from 9.83 to 11.65 (log_2_ expression units). Following LPS stimulation, *Srgn* expression was induced in most strains, and by more than 2-fold in 32% of the strains (Figure 1B). This observation led us to investigate whether the regulation of Srgn is instead linked to macrophage polarization, by examining correlations with established M1 and M2 markers (Figure 1C). As shown in Figure 1C, *Srgn* expression correlated positively with M1-associated (pro-inflammatory) markers and negatively with M2-associated (anti-inflammatory) markers. Hence, these findings suggest an association between serglycin and classical macrophage polarization into an M1-like phenotype. The list of genes used to assess M1 and M2 polarization is provided in Table S2.

To delineate regulatory networks linked to serglycin, we conducted co-expression analysis and identified genes with expression patterns significantly correlated with *Srgn*. This approach allowed us to uncover potential co-regulated genes and infer shared regulatory mechanisms that may contribute to the inflammatory response. In control (unstimulated) macrophages, pathways that correlated positively with *Srgn* expression were predominantly metabolic in nature, including mTORC1 signaling, oxidative phosphorylation, glycolysis, and fatty acid metabolism (Figure 1D). Pathways that were inversely associated with *Srgn* expression in control macrophages included cell cycle-related programs, such as E2F targets, G2M checkpoint, mitotic spindle, DNA repair, and MYC targets (Figure 1E). Although this analysis was performed in mouse macrophages, we further examined proliferation in human THP-1 macrophages using CellTiter-Glo and observed significantly reduced proliferation in serglycin knockout (SRGN-KO) cells. (Figure S1A). Together, these points toward *Srgn* expression being associated with highly metabolically active cells in a G0 state. Upon LPS stimulation, *Srgn* expression became strongly associated with pro-inflammatory pathways, including TNF-α signaling via NF-κB, interferon-γ (IFN-γ) and -α responses, inflammatory response, allograft rejection, and IL6/JAK/STAT3 signaling, all pathways pointing toward an immune activation (Figure 1F). Further, *Srgn* expression was negatively correlated with several mitochondrial and lipid metabolic pathways, including oxidative phosphorylation, heme metabolism, fatty acid metabolism, peroxisome activity, and cholesterol homeostasis (Figure 1G). This suggests that high Srgn expression is associated with a pro-inflammatory macrophage phenotype characterized by reduced oxidative phosphorylation.

### Serglycin deficiency modulates inflammatory signaling and macrophage activation

**Figure 2:**
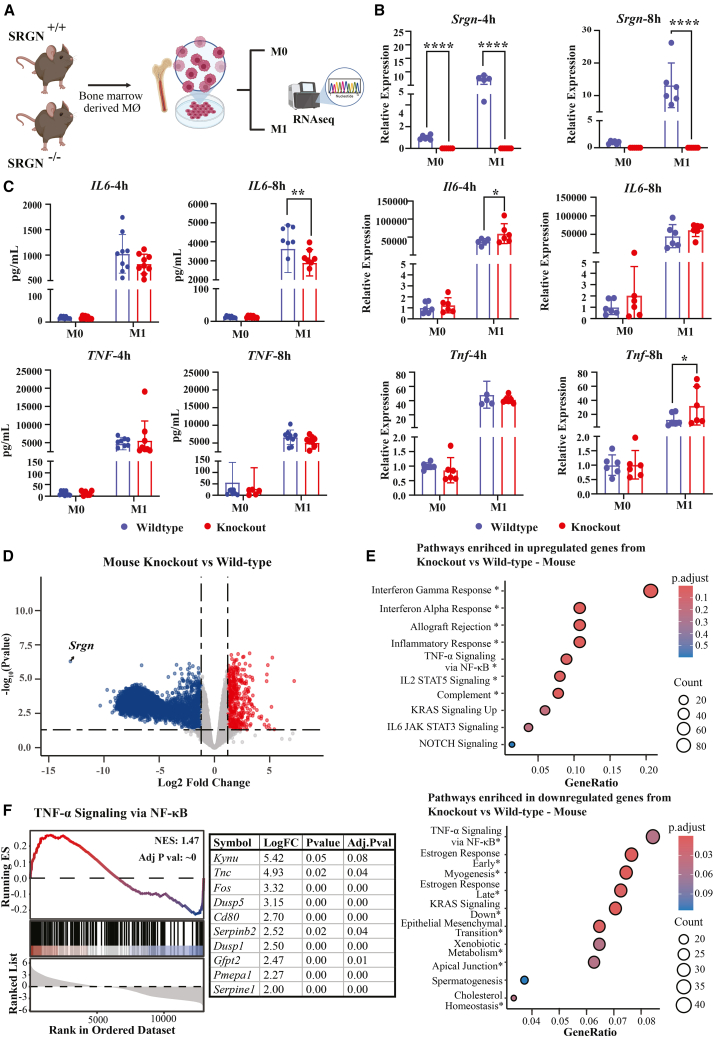
Loss of *Srgn* impairs pro-inflammatory signaling and cytokine production in macrophages (A) Experimental design. Bone marrow-derived macrophages (BMDMs) from wild-type (*Srgn*^+/+^) and knockout (*Srgn*^−/−^) mice were polarized to M1 with LPS and IFN-γ for 4 h or 8 h and subjected to RNA-seq and functional assays (*n* = 4). (B) Relative expression of *Srgn*, *Il6*, and *Tnf* mRNA at 4 h and 8 h of polarization. *Srgn* was absent in knockout BMDMs and significantly induced in wild-type cells under M1 conditions. *Il6* and *Tnf* were induced in both genotypes but reached significantly higher levels in *Srgn*^−/−^ BMDMs at 4 h (*Il6*) and 8 h (*Tnf*) (mean ± SEM; unpaired Student’s t test; *p* < 0.05, *p <* 0.01, *∗∗p <* 0.0001; *n* = 4). (C) Protein secretion of IL-6 and TNF at 4 h and 8 h. IL-6 secretion was significantly reduced in Srgn^−/−^ BMDMs at 8 h, whereas TNF secretion showed no significant differences (mean ± SEM; unpaired Student’s *t* test). (D) Volcano plot of differentially expressed genes in *Srgn*^−/−^ versus *Srgn*^+/+^ BMDMs at 4 h of M1 polarization. A total of 5,353 genes were significantly altered (fold-change threshold of 1.2, adjusted *p* < 0.05), with 1,206 upregulated and 4,147 downregulated in Srgn^−/−^ cells. (E) Hallmark pathway enrichment analysis of differentially expressed genes (fold-change threshold of 1.2, adjusted *p < 0.05*). Upregulated pathways in knockout BMDMs included interferon-γ response, interferon-α response, allograft rejection, inflammatory response, complement, and TNF-α/NF-κB signaling. Downregulated pathways included TNF-α/NF-κB signaling, estrogen response, KRAS signaling, epithelial-mesenchymal transition, and cholesterol homeostasis. Significant pathways are marked with an asterisk (∗). (F) Gene set enrichment analysis (GSEA) of TNF-α/NF-κB signaling, highlighting both upregulated and downregulated components in *Srgn*^−/−^ BMDMs. Representative upregulated genes (e.g., *Fos*, *Cd80*, and *Serpinb2*) are shown.

Although correlation analysis of RNAseq data can reveal co-expression patterns, it does not establish whether the genes of interest exert direct regulatory effects or act through immediate pathways. To gain mechanistic insight, we therefore examined serglycin’s contribution to M1 macrophage activation at early time points (4 h and 8 h), capturing the initial transcriptional phase before extensive secondary cytokine feedback occurs. BMDMs were isolated from serglycin-deficient (*Srgn*^−/−^)^4^ and wild-type (*Srgn*^*+/+*^) mice and remained untreated (M0) or were stimulated with LPS and IFN-γ for 4 and 8 h to induce M1 polarization (Figure 2A) and with IL-4 and IL-13 to induce M2 polarization (Figure S1B). Analysis of canonical M1 and M2 markers confirmed that SRGN-KO macrophages displayed attenuated induction of M1 genes but no shift toward an M2-like profile (Figure S1C). Because serglycin deficiency did not elicit notable transcriptional or phenotypic changes under M2 conditions, subsequent analyses focused on M1 macrophages, where the most prominent differences were observed. The particular focus on M1 polarization was based on the noted positive correlation between *Srgn* expression and M1 phenotype in the 92 investigated mouse strains (Figure 1C). As expected, *Srgn* expression was completely absent in the knockout BMDMs, while being significantly (*p* < 0.0001) induced in *Srgn*^*+/+*^ BMDMs under M1 polarization conditions (Figure 2B). Both the pro-inflammatory mediators *Tnf* and *Il6* were significantly induced under M1 conditions, as seen already at 4 h post initiation of the M1 polarization protocol, in both *Srgn*^*+/+*^ and *Srgn*^−/−^ BMDMs. However, *Tnf* and *Il6* were significantly higher expressed in the *Srgn*^−/−^ BMDMs as compared to the *Srgn*^*+/+*^ cells at 8 h and 4 h, respectively (Figure 2C). The difference observed in mRNA levels was not reflected at the level of secreted protein. Instead, both IL-6 and TNF secretion showed trended toward reduced in *Srgn*^−/−^ BMDMs compared to *Srgn^+/+^* cells at 4 and 8 h, with the decrease reaching statistical significance for IL-6 only at the 8-h time point (Figure 2C). These results indicate variability at early time points and should be interpreted as exploratory observations rather than definitive effects.

To globally assess the transcriptional effects of serglycin deficiency upon macrophage polarization, we subjected *Srgn*^*+/+*^ and *Srgn*^−/−^ BMDMs to M1 polarization (4 h), followed by RNA-Seq and differential gene expression analysis (Table S3). Following M1 polarization (Figure 2D), 5,353 genes were significantly differently expressed (adjusted *p*-value <0.05) between *Srgn*^*+/+*^ and *Srgn*^−/−^ BMDMs, with 1,206 genes being significantly upregulated and 4,147 genes being significantly downregulated in the absence of serglycin. Hence, the majority of genes that were differentially expressed between *Srgn*^*+/+*^ and *Srgn*^−/−^ BMDM showed downregulation in the knockout relative to the control. Hallmark pathway enrichment analysis of the differentially expressed genes revealed the upregulation of IFN-γ and interferon-α responses, allograft rejection, inflammatory response, and complement pathways in *Srgn*^−/−^ cells compared to *Srgn*^*+/+*^ (Figure 2E). Interestingly, TNF-α/NF-κB signaling emerged as both one of the most upregulated and most downregulated pathways in *Srgn*^−/−^ BMDM, highlighting its complex regulation by serglycin (Figure 2E).

GSEA of the TNF-α/NF-κB pathway revealed both up- and downregulated components, underscoring a complex regulatory pattern (Figure 2F and Table S4). Among the 179 genes in this pathway, 58 were significantly upregulated, and 56 were downregulated. *Il6* was among the most strongly induced genes, whereas *Tnf* showed only a modest but significant increase. Notably, serglycin-dependent effects on NF-κB-driven cytokine programs were already evident at 4 h of activation, well before the more commonly studied 24 h time point.

### Serglycin deficiency modulates inflammatory activation in human macrophages

**Figure 3:**
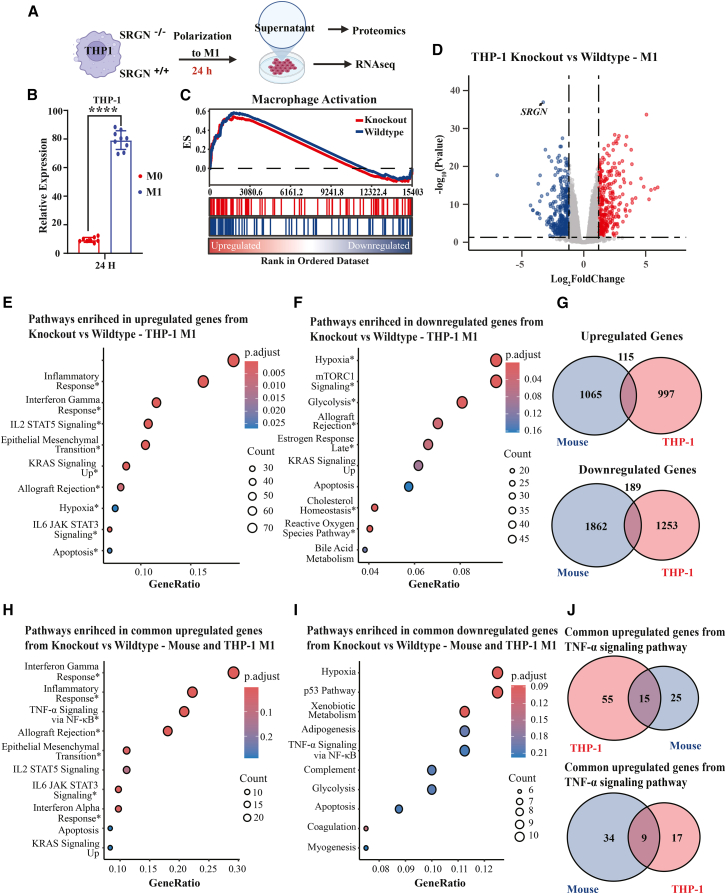
SRGN deficiency disrupts macrophage inflammatory activation and alters mitochondrial function (A) Experimental design. Wild-type (*SRGN*^+/+^) and knockout (*SRGN*^−/−^) THP-1 cells were differentiated into macrophages (M0) and polarized to M1 with LPS and IFN-γ for 24 h, followed by RNA-seq and proteomic analysis of culture supernatants (*n* = 4). (B) Relative *SRGN* expression in wild-type THP-1 cells, showing significant induction after M1 polarization (mean ± SEM; unpaired Student’s t test; ∗∗*p* < 0.0001). (C) Gene set enrichment analysis (GSEA) of the macrophage activation signature comparing *SRGN*^−/−^ and *SRGN*^+/+^ THP-1 cells. Knockout cells displayed a reduced extent of activation compared to wild-type cells. (D) Differential expression analysis (volcano plot) of *SRGN*^−/−^ versus *SRGN*^+/+^ THP-1 M1 cells. A total of 2,554 genes were significantly altered (adjusted *p* < 0.05, fold-change threshold of 1.2), with 1,112 upregulated and 1,442 downregulated in knockout cells. (E and F) Hallmark pathway enrichment analysis of significantly differentially expressed genes. Upregulated pathways in *SRGN*^−/−^ THP-1 cells included inflammatory response, interferon-γ response, interferon-α response, IL2/STAT5 signaling, and epithelial-mesenchymal transition (E). Downregulated pathways included mTORC1 signaling, glycolysis, cholesterol homeostasis, bile acid metabolism, and reactive oxygen species (ROS) pathways (F). Significant pathways are marked with an asterisk (∗). (G) Overlap of differentially expressed genes between *SRGN*^−/−^ THP-1 cells and *Srgn*^−/−^ murine macrophages. A total of 115 commonly upregulated and 189 commonly downregulated genes were identified. (H–I) Pathway enrichment analysis of commonly altered genes. Upregulated genes were enriched for inflammatory response and interferon pathways (H), whereas downregulated genes were enriched for TNF-α/NF-κB signaling and glycolysis (I). (J) Overlap of TNF-α signaling genes between human and mouse macrophages, revealing 15 commonly upregulated and 9 commonly downregulated genes. Notably, commonly downregulated genes included *ZFP36*, *KLF2*, *HES1*, and *BHLHE40*, regulators of cytokine production and inflammatory programs.

As described above, our initial analyses were performed in murine models, but validation in human systems is critical, given species-specific differences in immune responses and molecular pathways. To this end, we used the THP-1 human monocyte-like cell line, which can be differentiated into macrophage-like cells: M0 (basal; PMA for 24 h) and M1 (pro-inflammatory; LPS and IFN-γ for 24 h after M0 differentiation) (Figure 3A). Consistent with our findings in murine cells, polarization of THP-1 cells from M0 to M1 phenotype induced *SRGN* expression (Figure 3B). To directly examine its functional role, we generated serglycin-deficient THP-1 cells using CRISPR/Cas9, introducing a frameshift mutation. Wild-type and knockout M0 and M1 cells were subjected to RNA sequencing, and culture supernatants to proteomic profiling (Figure 3A). These analyses confirmed high *SRGN* expression in wild-type macrophages, with a significant increase upon M1 polarization, whereas *SRGN* expression was markedly reduced in knockout cells (Figure S2A). Following M1 polarization, both wild-type and knockout macrophages showed enrichment of inflammatory pathways accompanied by reduced expression of oxidative phosphorylation and cell cycle programs, consistent with classical macrophage activation (Figures S2B–S2E, Tables S5 and S6). By comparing the macrophage activation gene set using GSEA, we noted that the serglycin knockout (SRGN-KO) THP-1 cells displayed a slightly less extent of activation compared to wild-type cells (Figure 3C and Table S7). Among macrophage activation-associated genes, the largest differences between SRGN-KO and wild-type cells (M0 vs. M1 polarization) were observed in three categories. First, several upregulated genes were less induced in SRGN-KO macrophages, including *C1QA, FCGR3A, SLC11A1,* and *TLR3*. In contrast, other upregulated genes were more strongly induced in the knockout cells, such as *FCER2, IL31RA, IL6, LRFN5,* and *WNT5A*. Finally, several downregulated genes showed greater suppression in the knockout vs. wild-type cells, including *CST7, CX3CR1, FCGR2B, SUCNR1, SYK, and TREM2*. No downregulated genes were identified that were less reduced in the knockout cells.

When comparing M1-polarized SRGN-KO with wild-type macrophages, we found 2,554 genes to be significantly differentially expressed, with 1,112 genes being upregulated and 1,442 genes being downregulated because of serglycin-deficiency (Figure 3D and Table S8). Notably, several of the inflammatory pathways that were differentially expressed in *Srgn*^+/+^ vs. *Srgn*^−/−^ murine macrophages were also affected by serglycin deficiency in human cells, including TNF-α signaling via NF-κB, Inflammatory response, and IFN-γ response (Figure 3E). In contrast, the major pathways downregulated in SRGN-KO human cells were predominantly metabolic, including mTORC1 signaling, glycolysis, cholesterol homeostasis, and Bile acid metabolism (Figure 3F). Pathway analysis further revealed that the reactive oxygen species (ROS) pathway was downregulated in SRGN-KO THP-1 cells compared with wild-type controls (Figure 3F). The observed differences in enriched pathways between mouse and human cells may reflect either the longer stimulation period used for THP-1 cells (24 h vs. 4 h) or intrinsic species-specific differences. To directly compare the two contexts, we performed pathway enrichment analysis on genes differentially regulated by serglycin deficiency in both human and murine macrophages. This revealed 115 commonly upregulated and 189 commonly downregulated genes (Figure 3G). Pathway analysis showed that the upregulated set was enriched for inflammatory response pathways (Figure 3H), whereas the downregulated set was enriched for TNF-α signaling via NF-κB and glycolysis (Figure 3I). Notably, different components of the TNF-α signaling pathway were oppositely regulated, leading to both positive and negative enrichment in human and murine macrophages. GSEA of the TNF-α signaling hallmark further demonstrated fewer significantly downregulated genes in THP-1 cells (26 genes) compared with BMDMs (56 genes) (Figure S2F and Table S9). Examining the overlap between TNF-α signaling genes in the two systems revealed 15 genes upregulated and nine genes downregulated in both cell types (Figure 3J and Table S10). Of particular interest, the commonly downregulated genes included *ZFP36, KLF2, HES1, and BHLHE40*, all of which regulate inflammatory responses in macrophages by modulating cytokine production and signaling. *ZFP36* and *KLF2* act as repressors by destabilizing pro-inflammatory mRNAs and promoting anti-inflammatory programs, respectively, while *HES1* dampens chemokine-driven inflammation.^19^ In contrast, *BHLHE40* promotes cytokine production. Taken together, their collective downregulation suggests a re-wiring of macrophage inflammatory control programs, potentially altering the balance between pro- and anti-inflammatory responses.

### SRGN deficiency reshapes the inflammatory secretome and reduces the number of vesicles and phagocytosis in human macrophages

**Figure 4:**
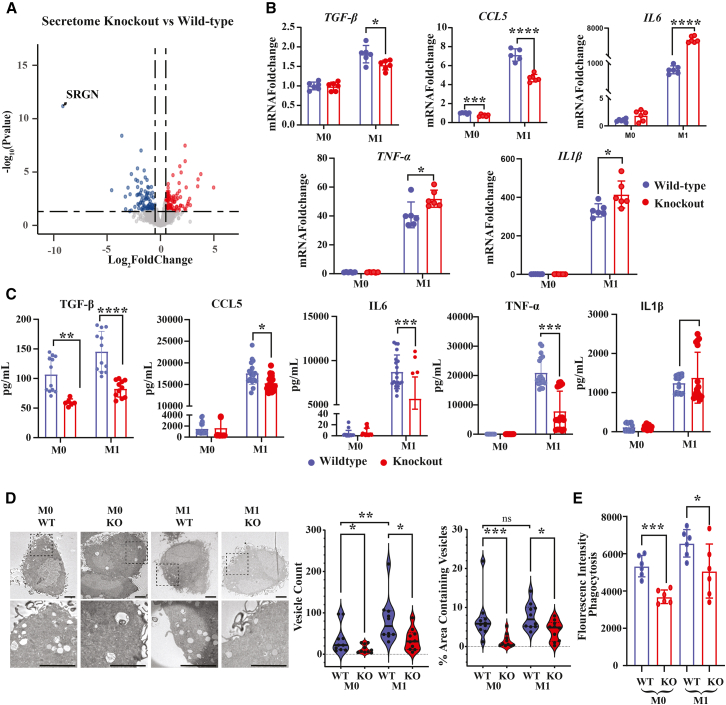
SRGN deficiency reshapes the inflammatory secretome and transcriptional programs in human macrophages (A) Volcano plot of differentially secreted proteins between *SRGN*^−/−^ and wild-type THP-1 macrophages under M1 polarization. Among 1,507 quantified proteins, 53 were significantly altered (adjusted *p* < 0.05), with serglycin being the most downregulated protein in knockout cells. (B) Validation of selected targets by RT-qPCR. *SRGN*^−/−^ M1 macrophages showed significantly increased expression of *IL6* and *TNF* and reduced expression of *CCL5* compared with wild-type cells (mean ± SEM; unpaired Student’s *t* test; *p* < 0.05, ∗*p* < 0.01, and ∗∗∗*p* < 0.0001; *n* = 4). (C) ELISA quantification of secreted cytokines in culture supernatants. *SRGN*^−/−^ macrophages secreted significantly less TNF-α, CCL5, and IL-6 compared with wild-type macrophages (*n* = 4), consistent with proteomics and RNA-seq data. (D) Transmission electron microscopy (TEM) images of THP-1 M0 and M1 macrophages. Scale bars, 5 μm. Vesicles were manually annotated and quantified in 10 cells per experimental group. The number of vesicles per cell and the percentage of cellular area occupied by vesicles were significantly reduced in both M0 and M1 *SRGN*^−/−^ macrophages compared with wild-type macrophages. (E) Phagocytosis assay using fluorescently labeled bioparticles. *SRGN*^−/−^ macrophages exhibited reduced phagocytic capacity under both M0 and M1 conditions (mean ± SEM; unpaired Student’s t test; *p* < 0.05 and ∗∗*p* < 0.001; *n* = 6).

Serglycin is known to be important for the storage and secretion of several inflammatory mediators in various cell types.^20^ To further examine how the secretion of proteins was affected by serglycin in macrophages, we analyzed the secretome of M1-polarized SRGN-KO and wild-type THP-1 macrophages by quantitative proteomics analysis of conditioned media. Among 1,507 quantified proteins, 53 were significantly differentially abundant in wild-type vs. SRGN-KO cells (adjusted *p*-value <0.05) (Figure 4A and Table S11). As expected, serglycin was the most downregulated protein in the secretome of knockout cells. Enrichment analysis revealed that proteins associated with IFN-γ and -α and TNF-α signaling via NF-κB (*SERPINB2, TNFAIP2, IL6, SERPINB8, TNFAIP8*) were upregulated in the secretome of SRGN-KO macrophages, i.e., in analogy with the findings from the RNA-Seq approach (Figure S3A). Intriguingly, and similar to the noted effects of serglycin deficiency on gene expression patterns, several proteins involved in TNF-α signaling (*CCL5, CSF1, SQSTM1, LDLR,* and *CCL20*) were also seen to be downregulated in the secretome of SRGN-KO THP-1 cells (Figure S3B).

To validate these findings by independent methodology, we performed RT-qPCR and ELISA analyses on selected targets in SRGN-KO and wild-type THP-1 macrophages. Upon M1 polarization, *IL6* and *TNF-α* were more strongly induced, whereas *CCL5* was less induced in SRGN-KO THP1 macrophages compared to WT cells (Figure 4B), consistent with the RNAseq data. Furthermore, ELISA measurements confirmed the quantitative proteomics analysis and showed significantly lower secretion of TNF-α and CCL5 in SRGN-KO THP1-macrophages compared to wild-type controls (Figure 4C). In contrast to the quantitative proteomics data from THP-1 cell media, ELISA measurements from THP-1 macrophage media revealed reduced IL-6 levels in SRGN-KO cells, consistent with the findings from murine BMDM media.

Macrophages are highly dynamic immune cells containing diverse vesicle populations that mediate phagocytosis, antigen presentation, intracellular trafficking, and secretion of cytokines and other mediators. Secretory vesicles are small, membrane-bound compartments involved in cytokine storage and release, whereas phagosomes are larger vesicles containing engulfed material, consistent with their role in pathogen degradation. To determine whether the altered secretome of SRGN-KO macrophages reflects changes in vesicular organization, we performed transmission electron microscopy of wild-type and knockout THP-1 macrophages under M0 and M1 conditions. SRGN-KO cells displayed a marked reduction in vesicle number, and the fraction of cytoplasmic area occupied by vesicles was diminished relative to wild type (Figure 4D). Vesicle-size distributions showed a uniform decrease across size categories, indicating a global impairment of vesicle biogenesis or maintenance rather than a selective effect on specific subpopulations (Figure S3C). In line with the structural defect, SRGN-KO macrophages exhibited significantly reduced phagocytic capacity in both resting (M0) and proinflammatory (M1) states; the reduction was most pronounced in M0 and remained evident under M1 polarization (Figure 4E).

Consistent with these morphological and functional defects, RNA-seq of SRGN-KO versus wild-type THP-1 cells revealed significant negative enrichment of the Gene Ontology term “regulation of vesicle-mediated transport” (FDR q < 0.05), with no vesicle-related GO terms positively enriched. In a cross-species comparison, genes commonly upregulated in SRGN-KO macrophages included *CCL2*, *LRRK2*, *NUMB*, *SDC4*, *SERPINE1*, whereas commonly downregulated genes included *CD300A*, *CD84*, *FCGR2A*, *FCGR2B*, *FCGR2C*, *GAS6*, and *LGALS3* (Figure 3J). Taken together, these morphological, functional, and transcriptomic data indicate a broader dysfunction in vesicle-dependent processes in SRGN-KO macrophages, consistent with serglycin’s role in granule organization and mediator trafficking.

### Serglycin ablation impacts mitochondrial respiration, lipid-driven metabolic flexibility, and ROS production in macrophages

M1 macrophage activation is known to involve metabolic reprogramming, including increased glycolysis, disrupted mitochondrial oxidative phosphorylation, and remodeling of lipid metabolism.^21^ Lipid metabolism shapes macrophage function by regulating lipid-mediated signaling, membrane dynamics, and inflammatory mediator biosynthesis. Notably, oleic acid, a monounsaturated fatty acid, has been reported to attenuate pro-inflammatory responses and alter lipid profiles during M1 polarization.^21^^,^^22^ In our study, the predominant pathways differentially regulated between WT and SRGN-KO macrophages were metabolic (Figures S3C and S3E). Oxidative phosphorylation was suppressed in both genotypes (THP1), but SRGN-KO macrophages showed additional downregulation of fatty acid metabolism, peroxisome activity, adipogenesis, and protein secretion, indicating a broader disruption of metabolic homeostasis. Since oleic acid metabolism depends on coordinated fatty acid oxidation, peroxisomal function, and oxidative phosphorylation, these findings suggest that SRGN deficiency impairs the ability of macrophages to process monounsaturated fatty acids, potentially altering lipid storage and inflammatory responses.

**Figure 5:**
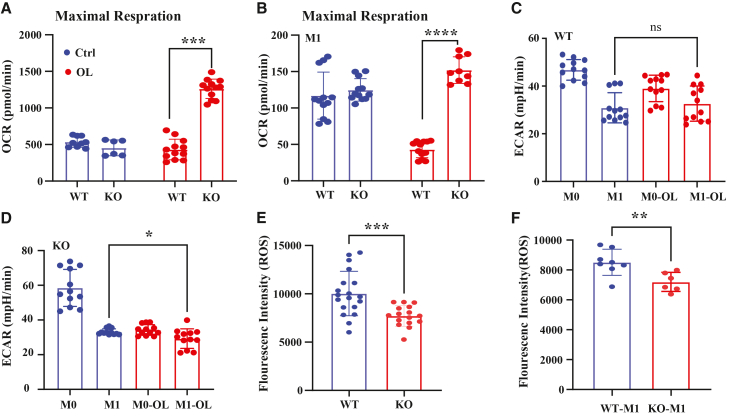
SRGN knockout alters the metabolic profile of macrophages (A and B) Oxygen consumption rate (OCR; maximal respiration) in wild-type (WT) and *SRGN*^−/−^ (KO) THP-1 macrophages under M0 (A) and M1 (B) conditions, with or without oleic acid (OA) supplementation. Oleic acid significantly increased mitochondrial respiration in *SRGN*^−/−^ macrophages (mean ± SEM; unpaired Student’s t test; ∗∗*p* < 0.001 and ∗∗∗*p* < 0.0001; *n* = 3). (C and D) Extracellular acidification rate (ECAR) in WT (C) and *SRGN*^−/−^ (D) macrophages under M0 and M1 conditions, with or without oleic acid. Oleic acid did not reverse glycolytic reprogramming in M1 macrophages; however, ECAR was altered in *SRGN*^−/−^ cells (mean ± SEM; *p* < 0.05; *n* = 3). (E and F) Reactive oxygen species (ROS) levels measured by DCFDA fluorescence. ROS levels were significantly reduced in *SRGN*^−/−^ macrophages compared with WT under both M0 (E) and M1 (F) polarization states (mean ± SEM; ∗*p* < 0.01 and ∗∗*p* < 0.001; *n* = 6).

To determine whether serglycin influences the metabolic shift during macrophage polarization, we measured oxygen consumption rates (OCRs) in WT and SRGN-KO THP-1 macrophages with or without oleic acid supplementation under M0 (Figures 5A and 5C) and M1 (Figures 5B and 5D) conditions. OCR decreased nearly 10-fold from M0 to M1 in both genotypes, consistent with the suppression of oxidative phosphorylation in favor of glycolysis. Oleic acid supplementation did not reverse this global reprogramming.

Notably, oleic acid supplementation markedly enhanced mitochondrial respiration in SRGN-KO macrophages under both M0 and M1 conditions (Figures 5A–5D), suggesting that serglycin constrains lipid-supported oxidative metabolism. Since mitochondrial ROS are generated as by-products of electron transport during OXPHOS, the reduced ROS levels observed in SRGN-KO macrophages (Figures 5E and 5F) are consistent with their impaired oxidative metabolism.

In M1 macrophages, the glycolytic switch with suppressed mitochondrial respiration typically promotes ROS production through succinate accumulation, reverse electron transport, and NADPH oxidase activity—processes required for pro-inflammatory and antimicrobial functions. However, in serglycin-deficient THP-1 macrophages, the ROS pathway was significantly downregulated at the transcriptional level (Figure 3F). Consistently, cellular ROS measured by the DCFDA assay were significantly reduced in knockout cells compared with WT controls under both M0 and M1 conditions (Figures 5E and 5F).

Together, these findings point to an association between serglycin deficiency and altered lipid-fueled mitochondrial respiration and redox balance in macrophages.

## Discussion

Our data support a role for serglycin as a conserved regulator of macrophage activation and secretory function across murine and human systems. In peritoneal macrophages from 92 genetically diverse mouse strains, LPS induced serglycin expression, linking it to canonical pro-inflammatory programs, including TNF-α signaling and interferon responses. Guided by these observations, we examined BMDMs from Srgn^−/−^ mice and found that, although the transcription of pro-inflammatory mediators such as TNF-α and IL6 was increased, secretion was often reduced. Consistent findings in CRISPR-Cas9-edited human THP-1 macrophages demonstrated that serglycin loss disrupts several M1-associated transcriptional responses, as revealed by integrated proteomic and transcriptomic analyses. Together, these findings suggest an association between serglycin expression and macrophage inflammatory output, which may be relevant for immune regulation and inflammatory disease.

A key observation was a divergence between transcription and secretion of proinflammatory cytokines. While mRNA levels of TNF-α were upregulated in serglycin-deficient macrophages, protein secretion was reduced in both *Srgn*^−/−^ murine macrophages and SRGN-KO THP-1 cells. Furthermore, proteomic analysis of the conditioned media from THP-1 macrophages revealed decreased CCL5, CSF1, and CCL20 and increased SERPINB2, TNFAIP2, and IL-6 in SRGN-KO cells. ELISA analyses on matched samples confirmed reduced secretion of IL6, CCL5, TGF-β, and TNF-α in SRGN-KO cells, indicating concordance for CCL5 but a discrepancy between the proteomic method and ELISA analysis for IL6. This discrepancy may reflect differences in assay chemistry and dynamic range (immunoassay vs. peptide-based MS), peptide detectability, and/or batch effects between experiments. Notably, many of the downregulated secreted proteins did not show corresponding decreases at the transcriptional level, whereas most upregulated secreted proteins were also transcriptionally upregulated. This pattern likely reflects serglycin’s role in facilitating protein secretion; in its absence, cells may compensate through feedback mechanisms that drive increased transcription of proteins normally regulated by this pathway. Additionally, we observed the dysregulation of proteins that regulate mRNA stability for inflammatory genes, which may account for the increased transcriptional expression of these genes. However, because we did not perform proteomic profiling of the cellular content in the knockout cells, we cannot determine which inflammatory proteins accumulate intracellularly as a consequence of serglycin loss, nor whether their secretion depends on a serglycin-mediated mechanism.

We have previously demonstrated that loss of serglycin results in reduced or delayed activation of epithelial-mesenchymal transition (EMT) in breast epithelial cells.^2^ Here, our findings suggest that the absence of serglycin also adversely affects macrophage activation. In murine macrophages subjected to 4 h of LPS stimulation, many genes were downregulated in the SRGN-KO macrophages, potentially reflecting a diminished or delayed inflammatory response mediated through the toll-like receptors. Additionally, M1 macrophage polarization was significantly less pronounced in THP-1 cells after 24 h of stimulation, as evidenced by a direct comparison of macrophage activation genes between SRGN-KO THP-1 and wild-type cells. Reduced metabolic shifts, decreased ROS production, and impaired phagocytosis further indicate a lower level of macrophage activation. Macrophages produce a complex variety of proteins—including cytokines (such as TNF-α, IL-1β, and IL-6), reactive species (including NO and ROS), and other signaling molecules—that play essential roles in their self-activation during the M1 activation process. This self-reinforcing mechanism is crucial for establishing a robust inflammatory response against pathogens and damaged tissues. However, excessive or prolonged activation can result in tissue damage and chronic inflammation.

Beyond transcriptional and secretory changes, serglycin loss also alters core aspects of macrophage biology. Transmission electron microscopy revealed that M1-polarized SRGN-KO macrophages contained fewer vesicles and a reduced vesicular area per cell, consistent with serglycin’s established role as a structural component of storage and secretory vesicles in immune cells^5^ and its necessity for histamine storage in mast cells.^23^ Together with our previous observation that serglycin-positive vesicles transiently expand following LPS stimulation in primary human monocytes,^13^ these findings implicate serglycin in vesicle biogenesis, trafficking, and secretion. This interpretation aligns with the observed discrepancy between transcript and protein secretion levels and parallels observations in mast cells from Srgn^−/−^ mice,^24^ suggesting a conserved role for serglycin in vesicle generation and turnover across immune cell types. Functionally, serglycin deficiency impaired phagocytic capacity, as indicated by reduced zymosan uptake and diminished reactive oxygen species generation, reflecting a broad attenuation of innate immune effector functions. Moreover, metabolic profiling revealed lower oxygen consumption and lipid oxidation in SRGN-KO cells, implicating serglycin in the coordination of inflammatory, phagocytic, and metabolic programs in M1 macrophages.

The present findings, obtained through three complementary experimental approaches, reinforce the concept of serglycin as a central mediator of inflammatory responses and, by extension, of pathologies in which macrophages and other serglycin-expressing immune cells play pivotal roles. Early work in serglycin-deficient mice established its role in regulating histamine and protease activity in mast cells, neutrophil elastase, and chemokines in platelets and macrophages, as well as in granule biogenesis.^3^ More broadly, serglycin deficiency has been linked to marked immune alterations, including splenic and Peyer’s Patch enlargement in aging mice,^11^ and to diverse pathological processes such as adipose tissue inflammation,^17^ intervertebral disc degeneration,^16^ osteoarthritis,^9^ dysregulation of serum LDL levels,^12^ complement pathway modulation,^25^ and tumor biology, including glioblastoma and other cancers.^26^^,^^27^^,^^28^ Given that chemokines are critical mediators of leukocyte recruitment and activation, and that their biological activity often depends on interactions with GAGs,^29^ our observation that CCL5 transcription and secretion are reduced in SRGN-KO macrophages—accompanied by impaired vesicle formation—underscores the functional relevance of serglycin both in immune regulation^8^ and in CCL5–GAG interactions during secretory vesicle biogenesis.^30^ Structurally, serglycin is a proteoglycan bearing covalently attached GAG chains, most commonly chondroitin 4-sulphate in immune cells, although macrophages and certain mast cell subsets synthesize highly sulphated chondroitin 4,6-disulphate, conferring greater binding affinity for partner molecules than chondroitin 4-sulphate, but lower than heparin.^3^ These structural features dictate serglycin’s capacity to bind chemokines, proteases, and other mediators, thereby shaping the amplitude and quality of immune responses. An important avenue for future research will be to determine how serglycin core protein expression, glycosyltransferases, and sulfotransferases are regulated—individually or coordinately—during immune activation. This remains largely unexplored^31^ but is likely central to understanding the functional diversity of serglycin in health and disease.

In cancer, the interplay between infiltrating immune cells, malignant cells, and the surrounding stroma is both complex and dynamic. Serglycin has been shown to promote EMT in breast cancer and is expressed by tumour-associated macrophages,^2^ suggesting multifaceted roles within the tumor microenvironment that may critically influence clinical outcome. The majority of breast cancers are estrogen sensitive, and macrophages likewise express the estrogen receptor, and upon activation by oestradiol or other ligands, these receptors reprogram macrophage transcription, signaling, and metabolism.^32^ SRGN-KO THP-1 cells did display reduced estrogen signaling by pathways analysis, a finding that was not followed up in this article. EMT can drive pathological fibrosis when tissue repair processes escape immune regulation, contributing to diseases such as lung and liver fibrosis and heart failure. In addition to its role in immune and inflammatory regulation, serglycin participates in tumour-stromal crosstalk across several cancers. In glioblastoma, its overexpression correlates with poor prognosis and elevated inflammatory mediators, TGF-β signaling components, CXCR-2, and proteolytic enzymes, collectively fostering tumor growth and fibroblast activation. This fibroblast reprogramming is dependent on CXCR-2 and TGFβRI signaling,^33^ a mechanism potentially relevant to fibroinflammatory pathologies, including hepatic fibrosis. Collectively, these observations expand the functional scope of serglycin beyond innate immune regulation to encompass the orchestration of stromal remodeling and EMT in both malignant and non-malignant disease contexts, highlighting the need for future studies to define its mechanistic contributions and therapeutic potential.

In atherosclerosis, plaque-infiltrating macrophages significantly contribute to lesion progression and stability.^34^ SRGN-KO macrophages exhibit reduced secretion of pro-inflammatory cytokines,^5^ which may diminish immune cell influx to atherosclerotic lesions and thereby curb inflammatory escalation within the plaque. Conversely, these same cells display an impairment in phagocytic capacity, potentially compromising the clearance of apoptotic foam cells—a process essential for anti-inflammatory resolution and plaque stabilization.^35^ The simultaneous dampening of cytokine release and phagocytosis may stem from a common mechanistic defect in vesicular trafficking: Serglycin, through its GAG side chains, acts as a scaffold that both organizes secretory vesicles and facilitates proper cargo loading. Its absence likely destabilizes vesicular structure and function, restricting cytokine secretion and impeding phagosome formation in parallel. Thus, rather than representing a contradictory phenotype, these effects reflect intertwined outcomes of disrupted serglycin-GAG-dependent vesicular regulation in macrophages, with potentially protective consequences in chronic inflammatory contexts such as atherosclerosis.

Together, these data identify serglycin as a modulator of macrophage plasticity, inflammation, and mitochondrial respiration, thereby integrating metabolic and immune functions upon inflammatory challenge. In chronic inflammatory settings such as cancer and atherosclerosis, where excessive macrophage activation perpetuates tissue injury, the absence of serglycin may help restrain pathological macrophage responses and promote a less inflammatory immune microenvironment, despite a concomitant reduction in phagocytic capacity.

### Limitations of the study

Data on serglycin function are limited to studies of cells cultured *in vitro*.

## Resource availability

### Lead contact

Requests for further information and resources should be directed to and will be fulfilled by the lead contact, Frode A. Norheim (f.a.norheim@medisin.uio.no).

### Materials availability

This study did not generate new unique reagents.

### Data and code availability

The RNA sequencing data generated in this study have been deposited in the NCBI BioProject database under accession number PRJNA1369785.

The mass spectrometry proteomics data have been deposited in the PRIDE repository via the ProteomeXchange Consortium under accession number PXD071481.

Code: All analysis scripts and code supporting the findings of this study are publicly available at GitHub (https://github.com/UiT-Tumorbiology/SRGN_Immuno).

Other: This study did not generate additional datasets or resources.

## Acknowledgments

We would like to thank Prof. Aldons Jake Lusis (UCLA) for giving us access to the HMDP macrophage dataset. We would also like to thank PRiME Proteomics and Metabolomics Core Facility (UiT) for their help with proteomic analysis and the Advanced Microscopy Core Facility (AMCF) (UiT) for help with the electron microscopy. The Figures are created with BioRender.com. The authors used ChatGPT to assist with grammar and clarity during the preparation of this manuscript. All content was subsequently reviewed and edited by the authors, who take full responsibility for the final text. Shirin Pourteymour has received funding from the 10.13039/501100007601European Union’s Horizon 2020 research and innovation program under the Marie Skłodowska-Curie grant agreement No 801133.

## Author contributions

F.A.N., E.K., G.P., K.T.D., and S.O.K. conceived and/or supervised the project. S.P., F.A.N., A.I.D., J.M., and E.K. conceptualized and designed the experiments. S.P., S.V.H., A.I.D., B.H., C.K.K., M.C., P.K., and C.D.B. performed the experiments. S.D.S., S.P., A.I.D., and C.K.K. conducted the data analyses. S.P. drafted the initial version of the manuscript. All authors read and approved the final manuscript.

## Declaration of interests

The authors declare no conflicts of interest.

## STAR★Methods

### Key resources table

**Table:** 

REAGENT or RESOURCE	SOURCE	IDENTIFIER
Hybrid Mouse Diversity Panel (HMDP) strains	The Jackson Laboratory	GEO: GSE38705
C57BL/6J mice	The Jackson Laboratory	RRID: IMSR_JAX:000664
Srgn−/− mice (C57BL/6J background)	Uppsala University	Dnr 5.8.18-02871/2023
THP-1 human monocytic cell line	ATCC	RRID: CVCL_0006
THP-1 SRGN knockout cells	This study	CRISPR/Cas9-generated
qPCR primers	This study	See Table S1
RPMI 1640 medium	Gibco	Cat# 61870036
DMEM	Gibco	Cat# 11065092
Foetal bovine serum (FBS)	Sigma-Aldrich	Cat# F7524-500ML
L-glutamine	Sigma-Aldrich	Cat# G7513
Penicillin–Streptomycin	Sigma-Aldrich	Cat# P0781
Lipopolysaccharide (LPS)	List Biological	Cat# 421
Lipopolysaccharide (LPS)	Sigma-Aldrich	Cat# L2630
IFN-γ	PeproTech	Cat# 300-02
IL-4 (recombinant human)	Sigma-Aldrich / Merck	Cat# SRP3093
IL-13 (recombinant human)	R&D Systems	Cat# 213-ILB
PMA	Sigma-Aldrich	Cat# P8139
Cas9 2NLS nuclease	Synthego	R20SPCAS9
Cell Line Nucleofector Kit V	Lonza	Cat# VCA-1003
High-Capacity cDNA Reverse Transcription Kit	Applied Biosystems	Cat# 4368813
SsoAdvanced Universal SYBR Green Supermix	Bio-Rad	Cat# 1725271
Human IL-6 ELISA kit	R&D Systems	Cat# DY206
Human IL-1β ELISA kit	R&D Systems	Cat# DY201
Human TNF-α ELISA kit	R&D Systems	Cat# DY210
Human CCL5 ELISA kit	R&D Systems	Cat# DY278
Human TGF-β ELISA kit	R&D Systems	Cat# DY240
Mouse IL-6 ELISA kit	R&D Systems	Cat# M6000B-1
Mouse IL-1β ELISA kit	R&D Systems	Cat# MLB00C
Mouse IL-12p40 ELISA kit	R&D Systems	Cat# M1270
Mouse TNF-α ELISA kit	R&D Systems	Cat# MTA00B
Seahorse XF Cell Mito Stress Test	Agilent	Cat# 103015-100
XF96 Cell Culture Microplates	Agilent	Cat# 101085-004
Seahorse XF Base Medium	Agilent	Cat# 102353-100
Oligomycin	Sigma-Aldrich	Cat# O4876
BAM15	MedChemExpress	Cat# HY-100558
Rotenone	Sigma-Aldrich	Cat# R8875
Antimycin A	Sigma-Aldrich	Cat# A8674
Lys-C protease	FUJIFILM Wako	Cat# 125-05061
Trypsin	Promega	Cat# V511A
DPX C18 tips	DPX Technologies	10 mg C18AQ 300Å
Acetonitrile (LC–MS grade)	Thermo Fisher Scientific	Cat# A955-4
Glutaraldehyde	Electron Microscopy Sciences	Cat# 15710
Osmium tetroxide	Electron Microscopy Sciences	Cat# 19110
Malachite green	Sigma-Aldrich	Cat# 101398
Tannic acid	Electron Microscopy Sciences	Cat# 21700
Uranyl acetate	Electron Microscopy Sciences	Cat# 22400
GraphPad Prism	GraphPad	v10
R	R Foundation	v4.5.1
edgeR	Bioconductor	v4.6.2
limma	Bioconductor	v3.64.1
MaxQuant	Max Planck Institute	v2.4.9.0
Fiji (ImageJ)	NIH	RRID: SCR_002285
THP-1 RNA-seq	NCBI BioProject	PRJNA1369785
Proteomics data	PRIDE via ProteomeXchange Consortium	PXD071481

### Experimental model and study participant details

#### Animals

The study included 92 mouse strains from the Hybrid Mouse Diversity Panel (HMDP), as previously described.^36^ Male mice aged 6–10 weeks were obtained from The Jackson Laboratory (Bar Harbor, ME, USA) and maintained under specific pathogen-free conditions on a standard chow diet (Ralston-Purina Co., St. Louis, MO, USA). Murine peritoneal macrophages were isolated from HMDP mice for downstream stimulation and transcriptomic profiling, as previously described^36^ and detailed in method details.

Bone marrow–derived macrophages (BMDMs) were generated from male Srgn^+^/^+^ and Srgn^-^/^-^ mice on a C57BL/6J background, bred and housed at the Swedish Veterinary Agency (Uppsala, Sweden). Animals were maintained under controlled environmental conditions with *ad libitum* access to food and water.

All animal experiments were conducted in compliance with institutional and international guidelines. Procedures involving the HMDP were approved by the Institutional Animal Care and Use Committee (IACUC) at the University of California, Los Angeles (UCLA) and conducted in accordance with the ARRIVE guidelines. Experiments involving Serglycin-deficient mice were approved by the Uppsala Animal Ethics Committee (approval no. Dnr 5.8.18-02871/2023) and conducted in accordance with EU Directive 2010/63 and relevant national animal welfare regulations.

Sex as a biological variable: Only male mice were used in this study; therefore, sex was not assessed as an experimental variable.

#### Cell lines

Human THP-1 monocyte-like cells (wild-type and SRGN knockout) were used for *in vitro* experiments. Culture conditions and differentiation/polarization protocols are described in method details.

Cell line authentication and routine contamination testing: Cell identity was consistent with the supplier’s characterization, and cultures were routinely monitored for morphology and growth characteristics. Mycoplasma contamination was periodically assessed using the MycoAlert™ Mycoplasma Detection Kit (Lonza, Cat. No. LT07-318) according to the manufacturer’s instructions, and all cultures used in experiments tested negative.

#### Human participants

No human participants or primary human samples were included in this study.

### Method details

#### Peritoneal macrophages

Cells were incubated with 2 ng/ml LPS (List Biological, Campbell, CA, Cat. No. 421) or a control in DMEM supplemented with 1% FBS for 24 hours prior to harvest. Total RNA was extracted using RNeasy columns (QIAGEN, Valencia, CA) with DNA digestion according to the manufacturer’s instructions and subsequently hybridized to Affymetrix HT MG-430A chip arrays. Chip signals were processed using robust multichip average (RMA) transformation. Raw microarray data for the peritoneal macrophages of HMDP was previously published and is available under NCBI GEO accession number GSE38705.

#### Generation of THP-1 knockout cells

Serglycin knockout cells were generated using CRISPR/Cas9-mediated genome editing as previously described. A single guide RNA targeting exon 1 of SRGN (sequence: 5′-CTG AGT CTT ACC TTG AAC TGA GG-3′) and Cas9 2NLS nuclease (R20SPCAS9, Synthego, California, USA) were delivered by electroporation using the Cell Line Nucleofector™ Kit V (VCA-1003, Lonza).^2^

#### Cell culture and differentiation

Human monocyte-like THP-1 cells (wild-type and SRGN knockout) and bone marrow–derived macrophages (BMDMs) were cultured in RPMI 1640 medium (Gibco, Cat. No. 61870036) supplemented with 5% foetal bovine serum (FBS; Sigma-Aldrich, Cat. No. F7524-500ML). THP-1 monocytes were differentiated into macrophage-like cells using 150 ng/mL phorbol 12- myristate 13-acetate (PMA; Sigma-Aldrich, Cat. No. P8139) for 24 hours, followed by a 24- hour recovery period in PMA-free medium. Cells were then polarized to either an M0 (resting) or M1 (proinflammatory) state by culturing in RPMI medium containing either vehicle (M0) or 20 ng/mL IFN-γ (PeproTech, Cat. No. 300-02) and 100 ng/mL LPS (Sigma-Aldrich, Cat. No.

L2630). M2 polarization was induced using 20 ng/mL IL-4 (Recombinant Human IL-4 Protein, Sigma-Aldrich/Merck, Cat. No. SRP3093) and 20 ng/mL IL-13 (Recombinant Human IL-13 Protein, R&D System, Cat. No. 213-ILB).

BMDMs were isolated from femur and tibia of male Srgn+/+ and Srgn-/- mice (C57BL/6J background), bred and housed at the Swedish Veterinary Agency (Uppsala, Sweden) as described (2). Bone marrow cells were flushed with PBS (27G needle), filtered (70 μm), and centrifuged (250 × g, 4 °C, 5 min). Erythrocytes were lysed using ACK buffer (Sigma-Aldrich), followed by washes in DMEM (Gibco, Cat. No. 11065092) supplemented with 15% L929 supernatant, 10% FBS, 4 mM L-glutamine (Sigma-Aldrich, Cat. No. G7513), 50 U/ml penicillin, 50 μg/ml streptomycin, Sigma Aldrich Cat. No. P0781). Cells were cultured in 15-cm dishes for 7 days, then seeded (5 × 10^5^ cells/ml) in 12-well plates. For polarization, cells were treated with 50 ng/ml LPS and 20 ng/ml IFN-γ (M1). Media and cells were collected after 4 and 8 hours for RNA extraction (QIAzol, Qiagen, Cat. No. 79306).

#### RNA isolation and qPCR

Total RNA was isolated using QIAzol Lysis Reagent (Qiagen, Cat. No. 79306) following the manufacturer’s protocol. RNA concentration and purity were assessed with a NanoPhotometer.NP80 (Implen, Germany). cDNA was synthesized from 500–1000 ng RNA using the High- Capacity cDNA Reverse Transcription Kit (Applied Biosystems, Cat. No. 4368813). Quantitative PCR (qPCR) was performed using SsoAdvanced Universal SYBR Green Supermix (Bio-Rad, Cat. No. 1725271) on a Bio-Rad CFX Duet Real-Time PCR System. Gene expression was normalized to GAPDH, RPLPO, and TBP, and relative levels were calculated using the 2−ΔΔCt method. The primer sequences are provided in Table S1.

#### Cytokine quantification by ELISA

THP-1: cytokine levels were measured using ELISA kits from R&D Systems: CCL5 (Cat. No. DY278), IL6 (Cat. No. DY206), IL-1β (Cat. No. DY201), TGF-β (Cat. No. DY240; activation kit DY010), and TNF-α (Cat. No. DY210), following the manufacturer’s instructions. Absorbance was recorded using a Synergy H1 microplate reader (BioTek). BMDMs: cytokine levels (IL6, IL-1β, IL-12p40, TGF-β1, and TNF-α) were measured in supernatants from differentiated BMDMs using ELISA kits (R&D Systems: IL6 [Cat. No. M6000B-1], IL-1β (Cat. No. MLB00C), IL-12p40 (Cat. No. M1270), TGF-β1 (Cat. No. DY1679-05, Cat. No. DY007B), TNF-α (Cat. No. MTA00B) following the manufacturer’s protocols. IL6 samples were diluted 1:40 to ensure detection within the standard curve range.

#### Cell proliferation assay (CellTiter-Glo)

Cell viability and proliferation were assessed using the CellTiter-Glo® Luminescent Cell Viability Assay (Promega, G7570), according to the manufacturer’s protocol. Luminescence was measured using a microplate reader (BioTek Synergy H1).

#### Phagocytosis assay (zymosan A beads)

Zymosan A bioparticles (Thermo Fisher Scientific Cat.No. Z23373) were used to assess phagocytic activity. THP1 cells were incubated with fluorescently labelled Zymosan beads at a bead-to-cell ratio of 5:1 for 2 hours at 37 °C. After incubation, non-internalized particles were removed by thorough washing and extracellular fluorescence was quenched with 0.2% Trypan Blue (Bio-Rad Cat. No. 1450021). Phagocytic uptake was quantified by measuring fluorescence intensity using a plate reader (excitation/emission: 495/519 nm).

#### Mitochondrial respiration analysis (seahorse XF cell mito stress test)

Mitochondrial respiration in wild-type and SRGN knockout (KO) THP-1 monocytes was assessed using the Seahorse XF Cell Mito Stress Test (Agilent, Cat. No. 103015-100) on the XF96 Extracellular Flux Analyzer. Cells were seeded at 80,000–100,000 cells/well in XF96 Cell Culture Microplates (Agilent, Cat. No. 101085-004), washed, and incubated for 1 hour in Seahorse XF Base Medium (Agilent, Cat. No. 102353-100) supplemented with 2 mM L-glutamine, 1 mM sodium pyruvate, and 10 mM glucose (pH 7.4) at 37°C in a non-CO_2_ incubator. Oxygen consumption rate (OCR) was measured under basal conditions and after sequential injection of 1.5 μM oligomycin (ATP synthase inhibitor, Sigma-Aldrich, Cat. No. O4876), 2.0 μM BAM15 (uncoupler, MedChemExpress, Cat. No. Hy-100558), and 0.5 μM rotenone/antimycin A (complex I/III inhibitors, Sigma Aldrich, Cat. No. R8875/A8674). OCR values were normalized to total protein using the BCA assay (Thermo Fisher, Cat. No. 23225) and analysed with Wave software (Agilent).

#### RNA-sequencing and transcriptomics

##### THP-1 cells (RNA-seq)

5 μg of DNase-treated total RNA was sent to Novogene (UK) Company Limited for library preparation and sequencing. Both wild-type and SRGN knockout samples were analyzed under M0 and M1 conditions after 24 hours in RPMI media, with six biological replicates per condition. Paired end 150 bp mRNA libraries were generated and sequenced on an Illumina NovaSeq 6000 platform. Raw FASTQ files were imported into CLC Genomics Workbench v22, quality-trimmed, and aligned to the human reference genome assembly GRCh38 with gene annotation from Ensembl release 104, as described previously.^37^ The resulting gene-level count matrix was exported to R (v4.5.1) for downstream analysis using R packages edgeR (v4.6.2) and limma (v3.64.1).^38^ Genes with less than 10 counts across all samples were removed using the filterByExpr function to reduce noise in downstream analysis. Normalization was performed with the trimmed mean of M values (TMM) method, and expression was converted to counts per million (CPM). Multidimensional scaling (MDS) analysis revealed replicate-specific batch effects, which were incorporated into the design matrix. MDS and sample correlation analysis further identified replicate 3 of the knockout M0 condition as an outlier. Differential expression analysis was conducted using the voom–lmFit pipeline, with the Treat function applied to enforce a minimum fold-change threshold of 1.2 for significance.

##### Bone marrow–derived macrophages (transcriptomic profiling)

AmpliSeq transcriptomic profiling was performed on LPS-stimulated mouse macrophages as described previously.^17^ Each condition included four biological replicates. Raw count data were preprocessed using the same pipeline as for the THP-1 RNA-seq dataset. Outliers were identified and excluded based on MDS and correlation analyses, leaving three replicates each condition. A minimum fold-change threshold of 1.2 was applied in the statistical testing.

##### Proteomics

5.0 × 10^6^ wild-type and SRGN-KO THP-1 cells were washed three times in PBS and resuspended in 10 ml serum-and phenol free RPMI 1640 (R7509 Sigma) for one hour. Cells were washed in PBS three additional times and cultured for 24 hours in 10 ml serum-and phenol free RPMI 1640 completed with 2 mM L-Glutamine (G7513, Sigma/Merck, Darmstadt, Germany) and 1% Penicillin-Streptomycin (P0781, Sigma/Merck). Cells were removed from media by centrifugation at 300 g for 5 minutes. The media containing the secretome was filtered through a 0.2μM sterile filter followed by 5 min centrifugation at 300 x g.The secretome was concentrated 50 times by centrifugation through a 3000MWCO Vivaspin 6(VS069, Sartorius, Goettingen, Germany) and washed twice with 10 mL milliQ water in the same filter. The final volume of the secretome was approximately 300 ul. The protein concentration was measured at 280 nm using Nanodrop ONE (Thermo Fisher Scientific, MA, USA). Aliquots of 30 μg protein were taken from each sample and evaporated dry in a SpeedVac. The dried pellets were resuspended in 40 μL buffer containing 100 mM ammonium bicarbonate and 1 mM CaCl2. Reduction of disulfide bridges was achieved by using 1,4-dithiothreitol (DTT) at a final concentration of 5 mM and incubation at 54°C for 30 min. Cysteines were alkylated with 15 mM iodoacetamide (IAA) and incubated for 30 min at room temperature in the dark. To remove excess IAA, DTT was added to a final concentration of 5 mM. Proteins were digested with Lys-C (125-05061, FUJIFILM Wako Chemicals Europe, Germany) at an enzyme to protein ratio of 1:100 for 5 hours at 37°C, followed by overnight digestion (16 hours) with trypsin (V511A, Promega, WN) at 1:20 enzyme to protein ratio under gentle agitation at 37°C. The next day, the samples were acidified using Trifluoracetic acid (TFA) and peptides were purified using DPX C18 pipette tips (DPX Technologies, XTR tips 10 mg C18AQ 300Å) on a Tecan Fluent pipetting robot. Purified peptide samples were dried in a vacuum concentrator and dissolved in 35 μL 0.1% formic acid. Peptide concentrations were measured on a spectrophotometer (Nanodrop ONE, Thermo Fisher Scientific, MA) at 205 nm. 0.5 μg of sample was injected for LC-MS analysis. Peptide mixtures containing 0.1% formic acid were loaded onto a Thermo Fisher Scientific EASY-nLC1200 system and EASY-Spray column (C18, 2 μm, 100 Å, 50 μm, 50 cm). Peptides were fractionated using a 5-80% acetonitrile (ThermoFisher Cat. No. A955-4) gradient in 0.1% formic acid over 60 min at a flow rate of 300 nL/min. The separated peptides were analysed using a Thermo Scientific Orbitrap Fusion Lumos mass spectrometer. Data was collected in data-dependent mode using a Top40 method using orbitrap detector for both MS1 and MS2. Raw MS data were processed in MaxQuant v2.4.9.0 against the human UniProt database. Proteins annotated by MaxQuant as “Reverse”, “Potential contaminant”, or “Only identified by site” were removed. In cases where UniProt IDs mapped to multiple gene names, unique identifiers were generated by concatenating the gene name with the corresponding protein ID. LFQ intensities were log2-transformed and structured according to the experimental design using the DEP R package.^37^^,^^39^ Proteins were retained if they were detected in at least two out of three replicates in one experimental condition. Missing values were imputed using the “manual” method (shift = 1.8, scale = 0.3) as implemented in DEP. Differential protein expression was analyzed using the limma framework with voom-like mean–variance modeling. Significance was determined using empirical Bayes moderated t-statistics with Benjamini– Hochberg correction for multiple testing. Because the number of quantified proteins was relatively small, the treat function was not applied for this dataset and no logFC threshold was applied for the statistical testing.

##### Transmission electron microscopy

THP-1 cells (wild-type and SRGN knockout) were seeded in 35 mm petri dishes and differentiated to M0 or M1 macrophages as described above. The cells were fixed with 4% formaldehyde and 1% glutaraldehyde (Electron Microscopy Sciences Cat. NO. 15710) in PHEM buffer. The cells were processed for TEM using 0.05% malachite green (Sigma-Aldrich, 101398), 1% osmium tetroxide (Electron Microscopy Sciences, Cat. No. 19110, Hatfield, PA)/0.8% K3Fe (CN)6 (Sigma-Aldrich, Cat. No. 702587), 1% tannic acid (Electron Microscopy Sciences, Cat. No. 21700), and 1% uranyl acetate (Electron Microscopy Sciences, Cat. No. 22400). This was followed by stepwise ethanol dehydration and embedding in epoxy resin (Agar, R1043, 1051,1081 and 1065). Due to THP-1 cells being suspension cells, centrifugation was performed between each preparation step. All processing steps were carried out using a microwave processor (Pelco BioWave, Ted Pella, Inc.). Finally, the resin was polymerized at 60°C for 48 h. Ultrathin sections (70 nm) were cut on a Leica EM UC7 using a 35° ultra-knife (Diatome) and collected on formvar coated copper grids. The cells were imaged using a Hitachi HT7800 transmission electron microscope. Images of ten arbitrary cells from each treatment group were used for quantification of vesicles. Vesicles were manually annotated and counted using Fiji (https://imagej.net/software/fiji/).

### Quantification and statistical analysis

#### Statistics analysis

All statistical analyses were performed using GraphPad Prism (version 10). Data are expressed as mean ± SEM. For comparisons between two groups, unpaired two-tailed Student’s t-test was used. One-way or two-way ANOVA followed by Tukey’s or Sidak’s post hoc test was applied for multiple group comparisons. Data normality was evaluated using the Shapiro–Wilk test. Correlation analyses were performed using Pearson’s or Spearman’s method, depending on the distribution. The number of biological replicates (n) for each experiment is stated in the figure legends. n represents independent biological replicates unless otherwise stated. Statistical significance was set at p < 0.05.

#### Differential expression and visualization

Differential expression results using the EnhancedVolcano R package.^40^ Venn diagrams of significantly upregulated or downregulated genes (adjusted p-value < 0.05) were generated with the VennDetail R package.^41^ For overlap analysis between THP-1 and mouse datasets, mouse genes were converted to their human orthologs using the Mouse Genome Informatics (MGI) resource.^42^

#### Functional enrichment analysis

Over-representation analysis (ORA) was performed on significantly upregulated and downregulated genes (adjusted p-value < 0.05) using the clusterProfiler package,^43^ with all expressed genes served as the background set. Hallmark and Gene Ontology (GO) databases were used as Liberzon et al., Gene Ontology Consortium et al.,^44^^,^^45^ with gene annotation provided by org.Hs.eg.db for human datasets and org.Mm.eg.db for mouse datasets. Gene set enrichment analysis (GSEA) was performed using clusterProfiler together with the msigdbr package.^46^ All expressed genes from the dataset were ranked by sign(logFC) × – log10(P.Value), and results were visualized using the GseaVis package.^47^

## Supplemental information

Document S1. Figures S1–S3

Table S1. Primer sequences used for mouse and human genes The table lists forward and reverse primer sequences employed for quantitative PCR analyses in mouse and human samples, respectively.

Table S2. Marker genes defining M(LPS)+ signature (M1) pro-inflammatory macrophages and M(LPS)− signature (M2) anti-inflammatory macrophages

Table S3. Differential gene expression analysis in M1 Srgn−/− versus Srgn+/+ bone marrow-derived macrophages

Table S4. Differential gene expression analysis of the TNF-α signaling via NF-κB hallmark gene set in M1 Srgn−/− versus Srgn+/+ bone marrow-derived macrophages

Table S5. Differential gene expression analysis in M1 versus M0 THP-1 serglycin wild-type cells

Table S6. Differential gene expression analysis in M1 versus M0 THP-1 serglycin knockout cells

Table S7. Differential gene expression analysis in M1 THP-1 serglycin knockout cells versus M1 wild-type cells

Table S8. Differential gene expression analysis of the Macrophage activation gene set in M1 versus M0 THP-1 serglycin wild-type cells and M1 versus M0 THP-1 serglycin knockout cells

Table S9. Differential gene expression analysis of the TNF-α signaling via NF-κB hallmark gene set in M1 THP-1 serglycin knockout cells compared to wild-type cells

Table S10. Overlap analysis of TNF-α signaling genes in M1 bone marrow-derived macrophages and M1 THP-1 cells

Table S11. Differential abundance analysis of secretome from M1 THP-1 serglycin knockout cells versus M1 wild-type cells
