# Supplementary material for: Serglycin modulates inflammation and metabolism in macrophages
Source: iScience. 2026 Mar 10;29(4):115235. doi: 10.1016/j.isci.2026.115235 (PMC13062519; doi:10.1016/j.isci.2026.115235)
Supplement: Document S1. Figures S1–S3 [file mmc1.pdf]

iScience, Volume *29*

Supplemental information

Serglycin modulates inflammation

and metabolism in macrophages

Shirin Porteymour, Saikat Das Sajib, Susannah von Hofsten, Atanaska I. Doncheva, Christina D. Bjørnvall, Beate Hegge, Mélina Gautier, Mirjana Grujic, Pratibha Kolan, Karthickeyan Chella Krishnan, Jason Matthews, Knut T. Dalen, Gunnar Pejler, Svein O. Kolset, Erik Knutsen, and Frode A. Norheim

Figure S1.Reduced proliferation in human SRGN knockout THP-1 macrophages.

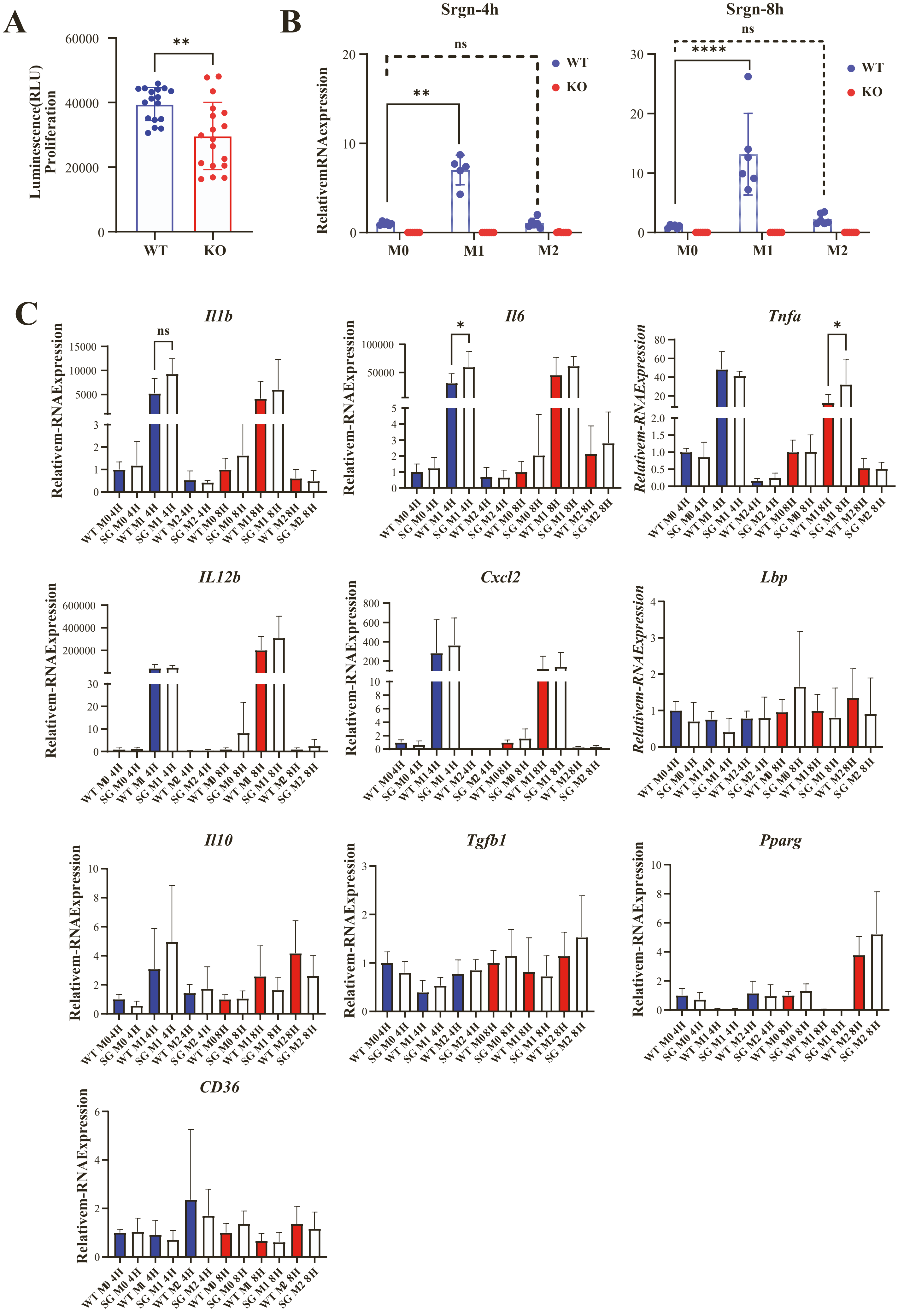

Figure S2. Serglycin deficiency modulates inflammatory activation in human THP-1 macrophages.

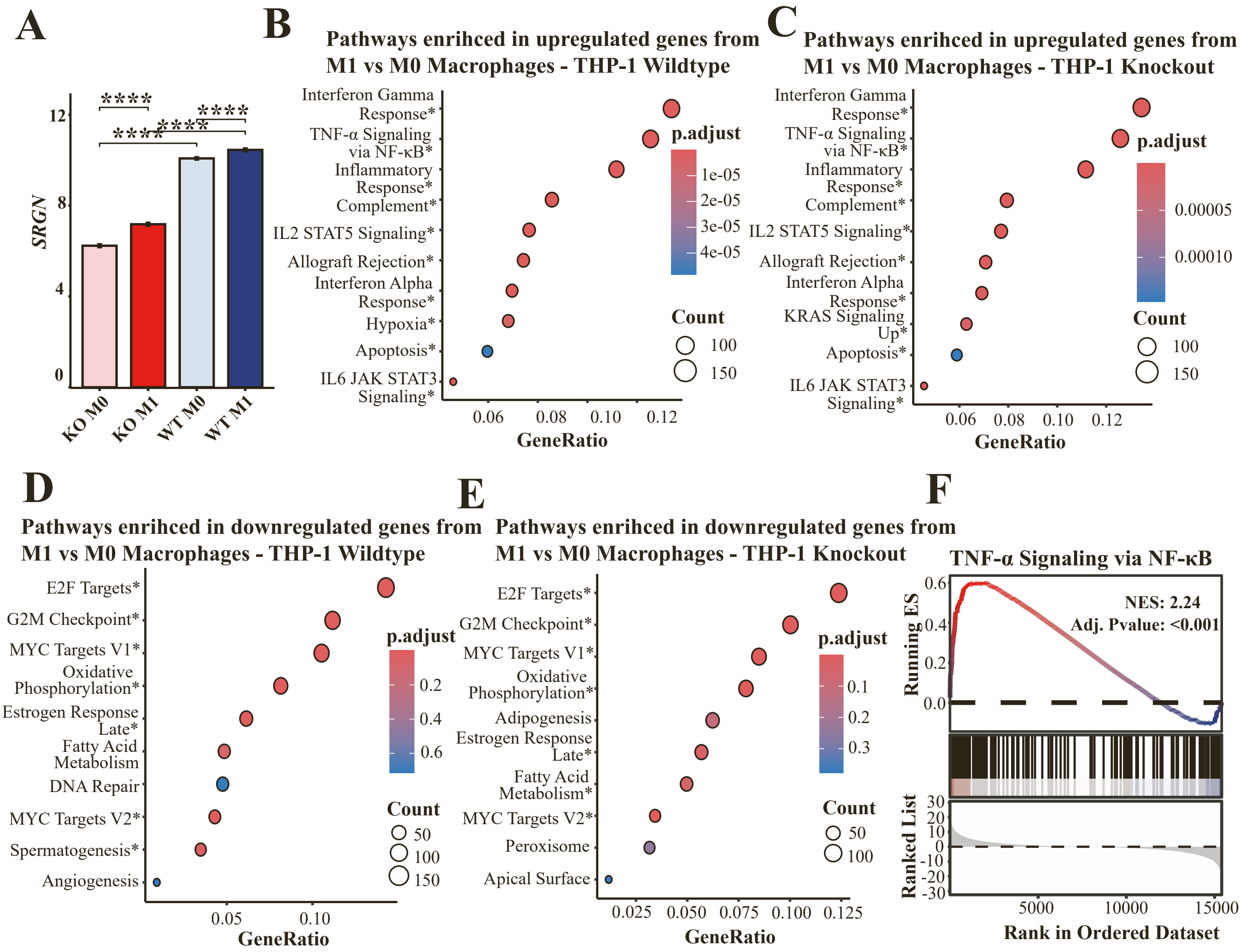

Figure S3. SRGN deficiency reshapes the macrophage secretome and vesicle architecture.

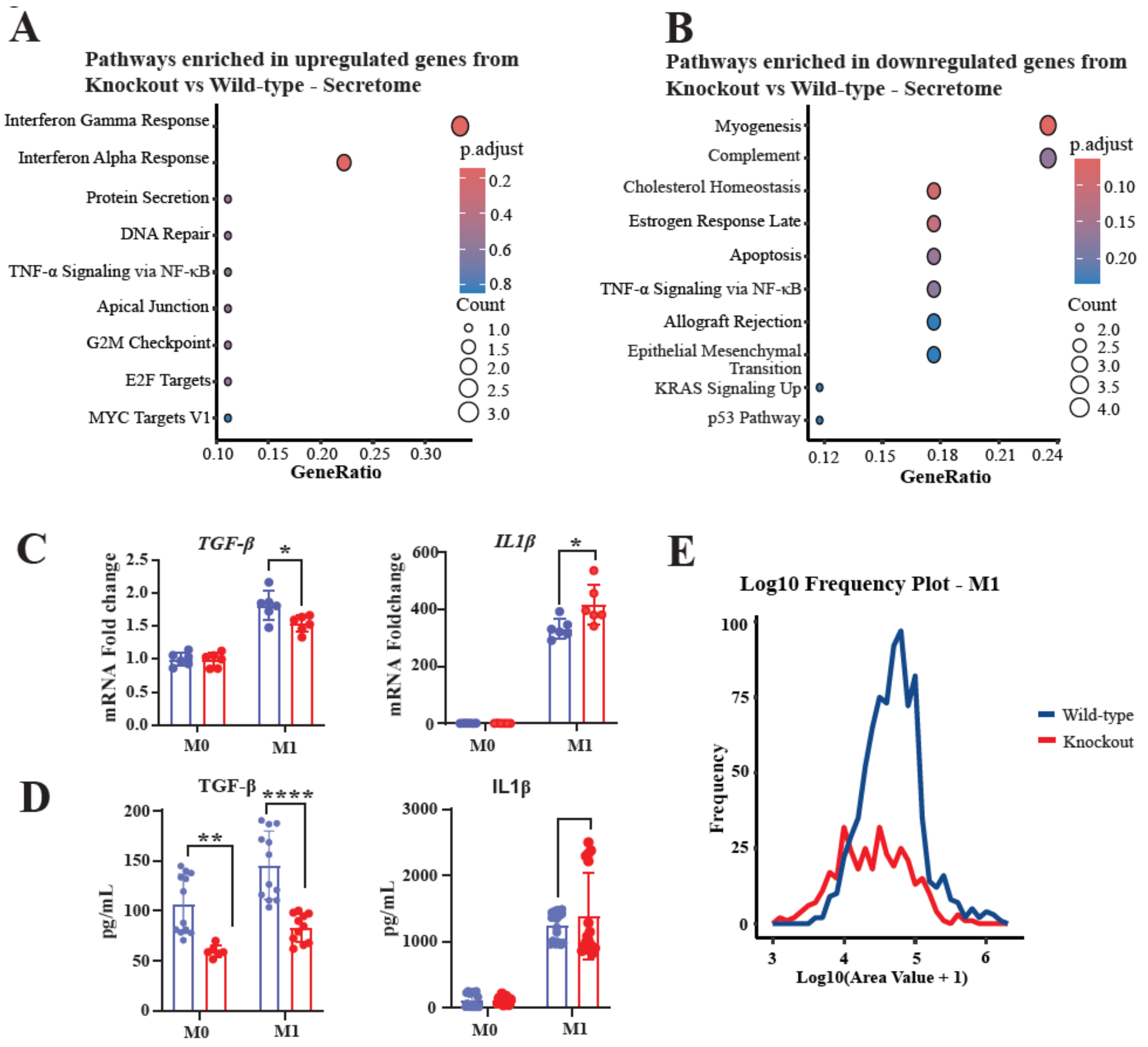

Figure S1. Reduced proliferation in human SRGN knockout THP-1 macrophages. (A) Human THP-1 monocytes (wild-type and SRGN knockout) were differentiated into macrophages and assessed for proliferation using the CellTiter-Glo luminescent assay. SRGN knockout cells exhibited significantly reduced proliferation compared to wild-type controls. Data are presented as mean ± SEM from n = 3 independent experiments. Statistical significance was determined using an unpaired Student's t-test (*p < 0.05, **p < 0.01, n=6). **(B) SRGN expression is selectively induced under M1 but not M2 polarization conditions**. BMDMs $Srgn^{+/+}$ and knockout $Srgn^{-/-}$ mice were left untreated (M0) or stimulated for 4 h or 8 h with LPS and IFN-γ (M1) or with IL-4 and IL-13 (M2). Srgn mRNA expression was markedly increased in WT macrophages under M1 conditions at both 4 h (p < 0.01) and 8 h (**p < 0.0001), while remaining unchanged under M2 conditions. No Srgn expression was detected in KO cells. Data are presented as mean ± SEM; unpaired Student's t- test; **n** = 4 biological replicates per group. (**C**) Expression of canonical M1 (Il1b, Il6, Tnf, Il12b, Cxcl2, Lbp) and M2 (Il10, Tgfb1, Pparg, Cd36) marker genes in BMDMs derived from $Srgn^{+/+}$ and $Srgn^{-/-}$ mice under basal (M0), M1 (LPS + IFN-γ), or M2 (IL-4 + IL-13) conditions at 4 h and 8 h. M1-associated genes were strongly induced in wild-type macrophages following LPS and IFN-γ stimulation but were attenuated in $Srgn^{-/-}$ cells. M2-associated genes showed minimal or no upregulation under IL-4/IL-13 stimulation, and no enhancement in $Srgn^{-/-}$ macrophages. Data represent mean ± SEM; unpaired Student's t-test; n = 4 biological replicates per group. M2 polarization was not strongly induced in WT BMDMs under the experimental conditions, at least at the mRNA level within the 4–8 h window.

Figure S2. Serglycin deficiency modulates inflammatory activation in human THP-1 macrophages. (A) *SRGN* expression in wild-type (WT) and knockout (KO) THP-1 macrophages under M0 and M1 conditions. *SRGN* was significantly induced in WT cells upon M1 polarization, whereas expression remained absent in KO cells (mean ± SEM; unpaired Student's t-test; ****p < 0.0001, n=4). **(B–C)** Pathways enriched in upregulated genes from M1 vs. M0 polarization in WT (B) and KO (C) THP-1 macrophages, showing enrichment of inflammatory programs including interferon-γ response, TNF-α signaling via NF-κB, and IL2/STAT5 signaling. Significant pathways are marked with an asterisk (*). **(D–E)** Pathways enriched in downregulated genes from M1 vs. M0 polarization in WT **(D)** and KO **(E)** THP-1 macrophages, including cell cycle–related programs, oxidative phosphorylation, fatty acid metabolism, and DNA repair. **(F)** Gene set enrichment analysis (GSEA) of TNF-α signaling via NF-κB in KO vs. WT THP-1 macrophages, revealing positive enrichment in KO cells (NES = 2.24, adjusted p < 0.001).

Figure S3. SRGN deficiency reshapes the macrophage secretome and vesicle architecture. (A–B) Hallmark pathway over-representation analysis (ORA) of differentially abundant proteins in the secretome of SRGN knockout (KO) versus wild-type (WT) THP-1 macrophages (M1, 24 h). Among 1,507 quantified proteins, 53 were significantly altered (adjusted $p < 0.05$). Upregulated proteins in KO cells were enriched for interferon-γ/α responses, TNF-α/NF-κB signaling, and pathways related to vesicle trafficking and protein secretion (A), whereas downregulated proteins were enriched for metabolic and stress-associated programs, including myogenesis, complement, cholesterol homeostasis, estrogen response (late), apoptosis, epithelial–mesenchymal transition, KRAS signaling (up), and the p53 pathway (**B**). Significant pathways are marked with an asterisk (*); *p.adjust* values are Benjamini–Hochberg–corrected. (**C–D**) *mRNA* and secreted protein levels of **TGF-β** and **IL-1β** in WT and SRGN-KO macrophages under basal (M0) and M1 conditions. *TGF-β* expression was modestly increased at both transcript and protein levels in SRGN-KO cells, while *IL1B* showed elevated mRNA but variable protein abundance (mean ± SEM; unpaired Student's *t*-test; * p < 0.05, ** p < 0.01, **** p < 0.0001; n = 4). **(E)** Frequency distribution of vesicle cross-sectional area from transmission electron microscopy (TEM) images of M1 macrophages (log10-transformed area + 1). SRGN-KO cells displayed a left-shifted distribution relative to WT, indicating globally smaller vesicle size.
